# A stepping stone to compositionality in chimpanzee communication

**DOI:** 10.7717/peerj.7623

**Published:** 2019-09-12

**Authors:** Linda S. Oña, Wendy Sandler, Katja Liebal

**Affiliations:** 1 Max Planck Research Group ‘Naturalistic Social Cognition’, Max Planck Institute for Human Development, Berlin, Germany; 2 Department of Education and Psychology, Freie Universität Berlin, Berlin, Germany; 3 Sign Language Research Lab, University of Haifa, Haifa, Israel

**Keywords:** Compositionality, Chimpanzees, Facial expressions, Gestures, Multimodality, Communication, Language evolution, Componentiality

## Abstract

Compositionality refers to a structural property of human language, according to which the meaning of a complex expression is a function of the meaning of its parts and the way they are combined. Compositionality is a defining characteristic of all human language, spoken and signed. Comparative research into the emergence of human language aims at identifying precursors to such key features of human language in the communication of other primates. While it is known that chimpanzees, our closest relatives, produce a variety of gestures, facial expressions and vocalizations in interactions with their group members, little is known about how these signals combine simultaneously. Therefore, the aim of the current study is to investigate whether there is evidence for compositional structures in the communication of chimpanzees. We investigated two semi-wild groups of chimpanzees, with focus on their manual gestures and their combinations with facial expressions across different social contexts. If there are compositional structures in chimpanzee communication, adding a facial expression to a gesture should convey a different message than the gesture alone, a difference that we expect to be measurable by the recipient’s response. Furthermore, we expect context-dependent usage of these combinations. Based on a form-based coding procedure of the collected video footage, we identified two frequently used manual gestures (stretched arm gesture and bent arm gesture) and two facial expression (bared teeth face and funneled lip face). We analyzed whether the recipients’ response varied depending on the signaler’s usage of a given gesture + face combination and the context in which these were used. Overall, our results suggest that, in positive contexts, such as play or grooming, specific combinations had an impact on the likelihood of the occurrence of particular responses. Specifically, adding a bared teeth face to a gesture either increased the likelihood of affiliative behavior (for stretched arm gesture) or eliminated the bias toward an affiliative response (for bent arm gesture). We show for the first time that the components under study are recombinable, and that different combinations elicit different responses, a property that we refer to as componentiality. Yet our data do not suggest that the components have consistent meanings in each combination—a defining property of compositionality. We propose that the componentiality exhibited in this study represents a necessary stepping stone toward a fully evolved compositional system.

## Introduction

Compositionality is an important characteristic of human language, which allows meaningful elements to be combined to create more complex structures (Werning, Hinzen & Machery, 2012). Morphemes combine to make complex words; words combine with other words in compounds and in phrases; phrases combine to make sentences; and simple sentences combine to form complex sentences. Given the building blocks and the rules at each level of structure, compositionality lends language a high degree of flexibility and productivity, as the combination of a limited number of components facilitates potentially open repertoires. Such flexibility is another key characteristic of human language (Jackendoff, 2011). Compositionality characterizes natural sign languages as well, demonstrating convincingly that all natural human language is compositional, whether produced by the voice and perceived by the ears, or produced by external bodily articulators and perceived by the eyes (Pfau, Steinbach & Woll, 2012; Sandler & Lillo-Martin, 2006). In sign languages, different articulators—the hands, the face, and the body—contribute to creating specific meanings, compositionally (Sandler, 2012).

Recent studies have proposed that compositionality is also found in other forms of human communication, in particular, in the expression of intense emotion. Human participants are able to distinguish emotions based on different configurations of the face and body (Cavicchio et al., 2018; Cavicchio & Sandler, 2015). This means that whole-body displays of emotions are composed of elements and sets of elements that contribute their meanings to the whole emotional display (Cavicchio et al., 2018; Sandler, 2018). The different elements of such a composite signal are conveyed by different articulators; for example, the signal can be a combination of a facial expression and a gesture, or a bodily posture and a facial expression, each contributing an aspect of emotional meaning. Based on these two types of visual communication in humans—sign language, and displays of intense emotion—Sandler (2018) concludes that humans are compositional communicators. Hearing speakers make ample use of visually perceivable gestures with speech (Goldin-Meadow & Singer, 2003; Kendon, 2004; Levy & McNeill, 1992; Müller, 2018). They also convey a panoply of facial expressions (Dols & Russell, 2017; Ekman & Friesen, 1978; Scherer & Ellgring, 2007; Swerts & Krahmer, 2008). We now understand, then, that language is not limited to vocal signals, but rather, that language is multimodal (Levinson & Holler, 2014; Müller et al., 2014; and see articles in Sandler, Gullberg & Padden (in press)).

In searching for the roots of human language, researchers adopting a comparative approach—comparing specific human traits with those of closely related species—focus on various properties of language, and investigate whether potential precursors are present in the vocal, gestural and facial communication of other primates. If we accept that human language (spoken or signed) is multimodal, it is essential that comparative research aiming to identify building blocks of language in other primates investigate their communication in an integrated, unified way. However, although Partan (1998, 2002) had already highlighted the importance of a multimodal approach two decades ago, the majority of comparative research into primate communication is still unimodal, focusing on the different signal types independently, without regard to their combination (Slocombe, Waller & Liebal, 2011). As a consequence, very little is known about the composition of structures that combine different signal types in primate communication.

This section “Compositionality and its Simultaneous Form in Visual Sign Languages” defines compositionality in language more precisely, and describes simultaneous visual compositionality in sign language. In the section “Chimpanzee Communicative Modalities,” we provide an overview of non-human primate signals in the context of compositionality. Our goal in the present study is to go beyond what is known, and to provide a model for investigating the ways in which chimpanzee signals combine. Our methodology follows in the “Materials and Methods” section. In the “Results” section we find that visual signals of chimpanzee communication are separable from one another, and that their combination can impact responses in recipients differently than their occurrence individually. We then summarize our findings in the “Discussion” section noting limitations of this preliminary study, and providing suggestions informed by it for future research.

## Compositionality and its simultaneous form in visual sign languages

We assume a simple basic definition for the term “compositionality”: “The meaning of a complex expression is a function of the meanings of its constituents (i.e., its parts/LO, KL,WS) and the way they are combined (Werning, Hinzen & Machery, 2012; p64),” where a meaningful “complex expression” is any expression with more than one indivisible meaningful part. For example, in English compound words, such as *law school*, the way in which the two words combine and their individual meanings reveal the meaning of the whole compound. The last word is typically the head or the anchor, supplying the basic meaning as well as the part of speech, while the preceding word further describes it in some way. In this example, the head is *school* and *law school* is a particular kind of school, one that is about law. The process of compounding in English has predictable properties. The meaning is typically derivable from the meanings of the two words individually, and the head is last. If we add more words, creating a more complex compound, such as *law school exam*, the head is *exam* and we are talking about an exam given at law school—and so forth: *law school exam application, law school exam application form*, etc. Any English speaker will automatically understand these new complex words because knowledge of their language includes knowledge of the meanings of the individual words and of the rules for combining them.

This principle stands behind the productivity of language at every meaningful level of structure, even inside the word. The suffix *-ness* is added to adjectives to create a new word having the quality of the adjective: *happiness, promptness, craziness*, etc. If speakers of a language encounter a new word with *-ness*, such as *savviness* (*savvy+ness)*, they will understand its meaning because they know the meaning of the parts and the rule for ordering them: -*ness* is added to the end of an adjective, changing it to a noun that has the quality of the adjective. Compositionality underlies our ability to produce and understand an infinite number of sentences as well. As such, it is a central property of human language. Here, we ask whether this property can be identified in other species.

The parts of a complex expression are typically sequenced linearly, as in the compound and complex words described above. However, the meaningful parts can also be combined simultaneously. The best examples of simultaneous combinations are found in sign languages used by deaf communities. Due to the physical production system of the hands, face and body, as well as to the visual perception system, simultaneous combinations are very common is sign languages generally (Sandler & Lillo-Martin, 2006). An example that is relevant to the present study involves combining meaningful manual signs with meaningful facial expressions. For example, to describe an event in which an entity is falling through space for a long time, signers of Israeli Sign Language combine the conventional sign meaning “fall” with a conventional facial expression meaning “for a long time.” These units are conventionalized and their combination is interpretable from the meanings of the parts. The same open-mouth facial expression occurring on any verbal sign will always mean “*verb* for a long time,” e.g., “convince for a long time,” “work for a long time,” etc.

Here, we ask whether this fundamental property of human language—compositionality—can be identified in other species, turning to our closest relatives, the chimpanzees. It is well known that chimpanzees possess relatively large repertoires of manual gestures and facial expressions (Hobaiter & Byrne, 2011). However, we do not know whether these signals combine and recombine compositionally. This study is a first step toward answering that question.

## Chimpanzee communicative modalities

Some studies that focus only on the vocal modality claim to provide evidence for compositional structures in call sequences, by showing that recipients respond differently depending on which calls are combined (Arnold & Zuberbühler, 2006, 2008; Clay & Zuberbühler, 2011; Ouattara, Lemasson & Zuberbühler, 2009a; Schlenker et al., 2016; Schlenker, Chemla & Zuberbühler, 2016). For example, Zuberbühler (2002) suggests that call combinations of monkeys are based on specific combinatory rules, and that the meaning of a call sequence can change depending on the order in which calls are combined. Alongside the scant evidence for compositional structure in the vocal domain, no evidence for compositional structures in gesture sequences has been found (Genty & Byrne, 2010; Liebal, Call & Tomasello, 2004; but see Tanner & Perlman, 2017; Tempelmann & Liebal, 2012), and facial expressions have not even been investigated in this regard. This might be at least partly due to the fact that facial expressions can occur in the form of “blended” signals consisting of two *simultaneously* produced gestures of the face (Parr, Cohen & De Waal, 2005), which makes it difficult to separate their individual components. This is even further complicated by the fact that facial expressions are often inseparably linked to the production of vocalizations, such as screams, which typically co-occur with a bared teeth display. Thus, in the vocal modality, there is limited evidence for compositionality, and in the visual modality alone (facial and gestural signals), there have as yet been no studies of compositionality in non-human primate communication.

Only more recently, the first studies of combinations comprising different signal types (vocal and gestural) have emerged, although the studies did not investigate compositionality in these combinations. Hobaiter, Byrne & Zuberbühler (2017) investigated gestural and vocal combinations in wild chimpanzees, who used such combinations in positive (affiliative) and negative (agonistic) interactions often involving the highest-ranking male. Chimpanzees switched to gesture-vocalization combinations if their unimodal communicative attempt was unsuccessful, but only if the initial signal was a vocalization. Wilke et al. (2017) focused on response rates to gesture-vocalization combinations compared to response rates to their individual components in wild chimpanzees. They found that the rate at which the recipients responded to gesture-vocalization combinations was similar to the rate at which they responded to single gestures, but not to their vocal components, where the response rates were lower (Wilke et al., 2017).

Although these studies suggest that chimpanzees flexibly use and respond to combinations of different signal types, it is currently unclear whether such multimodal combinations are compositional structures.

The determination of meaning is hampered by the fact that researchers differ drastically in terms of whether and how they assign specific meanings to signals across gestural, facial and vocal studies (Liebal & Oña, 2018). For example, vocal research has largely focused on whether primate calls have specific referents (such as specific types of predators or food), determined by the reactions of the recipients of these calls, while in the visual modalities, such as gestures and facial expressions, receiver reactions have been investigated to a much lesser extent (but see Hobaiter & Byrne, 2014). Unlike vocalizations, primate gestures have been commonly characterized by their “contextually defined usage” (Pollick & De Waal, 2007), as a single gesture can have different meanings depending on the contexts in which it is used. These differences in conceptual and methodological approaches across signal types have hampered multimodal approaches, and as a result, studies investigating potential compositional structures in chimpanzee communication are currently lacking.

The aim of our study is to investigate components of chimpanzee communication, with a focus on multimodal combinations of gestures and facial expressions. While vocalizations are undoubtedly crucial in chimpanzee communication, we focus only on visual displays in this study. There are two reasons for this approach. First, focusing exclusively on visual signals simplifies what is a very complex problem at this preliminary stage of investigation of the issues that interest us. Second, we know from sign language research sketched above that visual signals alone, e.g., of the hands and of the face, can enter into compositional structures.

To grapple with the task of determining a signal’s meaning, we used a *form-based approach* informed by sign language studies, which propose that actions of the hands, facial features, the head and the body each lend meaning to composite forms (Sandler, 2012, 2018). However, identifying the intended meaning of the signaler for each signal type may be challenging, as each signal type can convey various meanings (Hobaiter & Byrne, 2014). Far be it from us to define “meaning” in chimp signals in a way that would satisfy philosophers or semanticists. Instead we assume that meaning can only be interpreted based on context of use and response of recipients.

Therefore, we investigated whether the response behavior of the recipient varied depending on the signaler’s usage of gesture + face combinations and the context in which these expressions were used. Although it is true that much of the gesture work focuses on the context of use, it is often less well known that gesture researchers have also analyzed the response to specific gestures (Liebal, Pika & Tomasello, 2006). “Context” is always rather broad and it is challenging to code it properly, as clear-cut distinctions are sometimes difficult to make and the context might also change during an ongoing interaction—e.g., if play gets too rough, this might change into an aggressive interaction. Our approach aims to consider as many variables as possible to investigate exactly which behavioral aspect changes when a gesture is combined with a facial expression; thus, both the context and the recipient’s response are included in our analyses.

If, in a certain context, a gesture alone elicits a different response compared to the gesture + face combination, we surmised that the facial expression contributes an element of meaning. This would in turn indicate that the components of chimpanzee communication are not conveyed and interpreted as holistic signals, a first step toward compositionality. If a particular facial expression affects a different gesture in the same way (just as -*ness* always indicates a quality regardless of the word it attaches to, and as the open mouth facial expression in our sign language example always indicates duration regardless of the verb it occurs with), then this would indicate the existence of compositionality. We focused exclusively on a detailed analysis of two variants of the “extend arm” gesture (either with the whole arm stretched or with the hand or lower arm bent) and its combinations with different facial expressions in chimpanzees.

## Materials and Methods

### Study site and subjects

Observations were conducted at Chimfunshi Wildlife Orphanage Trust (Chimfunshi), a chimpanzee sanctuary accredited by the Pan African Sanctuary Alliance (PASA). Chimfunshi is located in the Copperbelt region of Northwestern Zambia, approximately 60 km west of Chingola on the southern bank of the Kafue River. Chimfunshi provides shelter for more than 120 chimpanzees, who live in four groups with large outdoor enclosures in the densely vegetated Miombo forest. We observed two groups of chimpanzees with a total of 72 individuals. Group 1 inhabits a 190-acre enclosure and group 2 inhabits a 160-acre enclosure. Each group comprises a mix of wild-born individuals, who were rescued from illegal trade, and sanctuary-born individuals. Group 1 consisted of 23 individuals (10 adult males, eight adult females, one juvenile male, two juvenile females, one infant male, one infant female). Group 2 consisted of 49 individuals (11 adult males, 28 adult females, five juvenile males, one juvenile female, one infant male, two infant females). As data collection took place in two consecutive years, group compositions slightly varied due to new-born infants (*N* = 3) and the death of individuals (*N* = 2).

### Ethical note

Our method of data collection was purely observational, with no changes necessary in the chimpanzees’ daily routine. This research was evaluated and approved by the Chimfunshi Research Advisory Board, a local ethics committee consisting of members of the management, researchers, and veterinarians (Chimfunshi Research Advisory Board no. #2014C016). The research strictly adhered to the legal requirements of Zambia (Zambia Wildlife Authority) and the PASA Primate Veterinary Healthcare Manual.

### Data collection

Data collection took place between June and August 2015 and June and August 2016, between the hours of 9:00 and 17:00. The chimpanzees are always outdoors except during feeding time, between 11:30 and 13:30, which they can (but do not have to) spend inside an indoor enclosure. A second feeding takes place outside in the afternoon, between 13:30 and 15:30. Because of this daily routine, data collection peaked in the hours before and after feeding, as chimpanzees were then close to the building, enabling us to observe and video-record their behavior.

**Figure 1:**
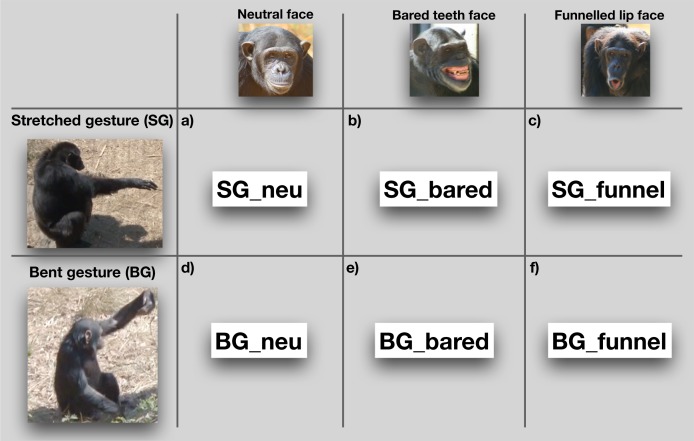
Overview of the different elements of the gestures and facial expressions (including neutral face) and the possible combinations. The stretched arm gesture (SG) as (A) a unimodal gesture; and in combination with (B) bared teeth face (SG_bared) and (C) funneled lip face (SG_funnel). The bent arm gesture (BG) as (D) unimodal gesture; and in combination with (E) bared teeth (BG_bared) and with (F) funneled lip face (BG_funnel).

We conducted pilot observations to establish the chimpanzees’ gestural repertoires. Using a form-based approach, we identified two frequently occurring variants of a manual gesture pictured and described in Fig. 1 below: the stretched arm gesture (SG) and the bent arm gesture (BG). These two variants have been described in previous studies (Roberts et al., 2012); however, there is no consensus and no systematic study about what they mean and what their function is. We followed the approach by Bard et al. (2019), and focused on two gesture types only, as this enabled us to conduct a detailed analysis of how these gestures were flexibly used to achieve varying social goals. Pilot observations also showed that SGs and their combinations with facial expressions mostly occurred in interactions involving the high-ranking males (as found by Hobaiter, Byrne & Zuberbühler, 2017), when they were arriving at the indoor enclosure. This resulted in a variety of positive (e.g., greeting, grooming) and negative (e.g., physical conflicts) interactions, in which the two gesture types were frequently used. Therefore, we conducted all occurrence recordings with focus on the two highest ranking individuals of groups 1 and 2, respectively. If these individuals were not visible, we focused on other high-ranking individuals in situations likely to involve the use of the two gesture types. The information about the individuals’ ranks was provided by the animal keepers and the veterinarian. Observations were conducted from the roof of the chimpanzees’ indoor enclosure (height: 5.5 m) or in front of the fence near this building. Since the chimpanzees often moved during filming, the observer held the camera so as to be able to adjust the position quickly if necessary. On the rooftop, the observer’s movement was restricted to one to three m, while in front of the building, the observer could move between 1 and 20 m depending on the enclosure. However, while filming an event, the observer tried to keep as still as possible and only moved when necessary. The chimpanzees were observed for a total of 600 h, resulting in 65 h of video material, recorded with a Sony Handycam HDR-FX1000E.

### Coding

**Figure 2:**
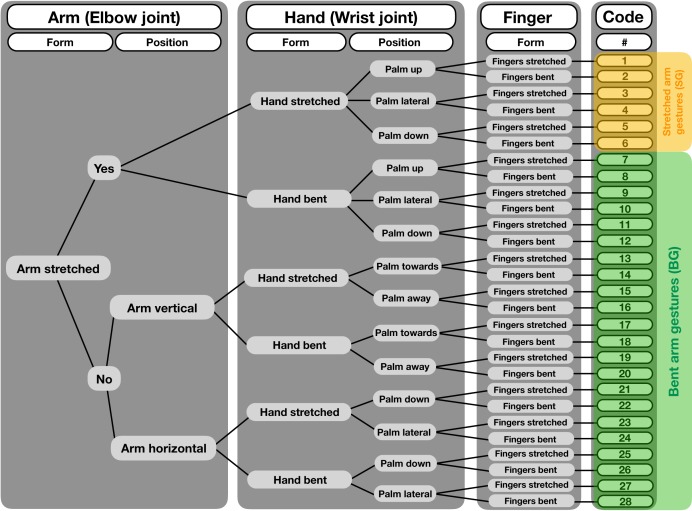
Decision tree for a structure-based gesture coding regime. Starting from left, decisions for each gesture event are made regarding form and position of the different articulators, i.e., arm (elbow joint), hand (wrist joint) and fingers. After following each step to the right, codes are given to the single gesture forms, totaling 28 different gestures. For our analysis, we grouped the different gestures into two categories, the “bent arm gesture” (BG) and the “stretched arm gestures” (SG).

We coded gestures and co-occurring facial expressions in dyadic interactions. For gesture coding, we first conducted pilot observations (ad libitum sampling on site for 10 days in June 2015 before the data collection started, in addition to screening older video footage collected by K.L. in June 2013 and June 2014) to examine when individuals would be visible, and to identify instances of gestural communication and the contexts in which they frequently occur. We used a form-based coding scheme, with a tree-like decision structure (see Fig. 2), which considered different morphological variants based on movements of the joints of the arm, hands and fingers. In this way, we differentiated 28 potential gesture types. However, some of these variants never occurred or were observed too rarely. Therefore, we grouped the different gestures into two variants—SG and the BG. The SG gesture, shown in Figs. 1A–1C, consists of an extended arm with both the arm and hand stretched. It has also been described as “reach” or “extend arm” gesture in both captive and wild chimpanzees (Hobaiter & Byrne, 2011; Liebal, Call & Tomasello, 2004). It is used in various contexts and mainly results in affiliative behaviors (e.g., individuals move closer together), such as grooming or food-sharing (Hobaiter, Byrne & Zuberbühler, 2017; Luef & Pika, 2017; Roberts et al., 2012). Second, for the BG gesture, part of the extended arm—either the hand or the forearm—is bent, in a way that the back of the hand or the forearm are directed toward the recipient (Figs. 1D–1F). This gesture has been previously described as “wrist offer” (Liebal, Call & Tomasello, 2004; Tomasello et al., 1997), but not much is known about its social function. The BG gesture is akin to what Roberts et al. (2012) describe as the “hand bend” gesture, which chimpanzees used during greeting and in submissive contexts, and the authors suggest that it elicits affiliative behavior and/or the cessation of antagonistic arousal (Roberts et al., 2012)^1^1Roberts et al. (2012) mentioned a similar gesture (“present arm”), which consists of up- and down movements. However, to our knowledge, the function of this gesture has not been described and therefore it is not clear whether it occurs in specific contexts or leads to specific responses of the recipient.. We observed other manual gestures (see gesture coding scheme in Supplemental Material), however, as noted, they occurred too rarely to include them into our analyses.

Facial expressions were coded by a coder (L.O.) experienced both with the human Facial Action Coding System (FACS; Ekman & Friesen, 1978) and with its modified versions for other species including chimpanzees (ChimpFACS; Vick et al., 2007). This standardized, objective method enables researchers to identify and classify facial expressions based on a defined set of minimal facial movements.

Two types of facial expressions were identified based on the prototypical chimpanzee facial expressions described by Parr, Waller & Vick (2007), which frequently occurred in combination with these gestures^2^2We observed other facial expressions (e.g., pout face), but their frequency of occurrence was too low to be included into our analyses.. First, the “bared teeth face,” shown in Figs. 1b and 1e, consists of the mouth either slightly opened or closed, the lips withdrawn and mouth corners retracted laterally, while the teeth are fully exposed (Parr, Cohen & De Waal, 2005). Although this expression is often described as an affiliative signal, it has been observed in a range of social contexts (Van Hooff, 1973; Waller & Dunbar, 2005), suggesting that it might have various functions in chimpanzees. Second, the “funneled lip” facial expression (Fig. 1) consists of a rounded and open mouth, and protruded lips. Van Lawick-Goodall (1968; p.88) described the expression as “pouted trumpeted lips that go with pant-hooting. The mouth may open completely or partially.” This facial expression is inherently linked to the production of hoot (or pant-hoot) vocalizations in chimpanzees. Pant-hoots occur in various situations that include general excitement, such as distress or bluff displays (Parr, Cohen & De Waal, 2005; Van Hooff, 1973), as part of ritualized agonistic displays of adult male chimpanzees (Mitani et al., 1992), or upon arrival at an abundant food source (Notman & Rendall, 2005), suggesting that it might have various functions. Whenever a gesture occurred without any facial expression, we coded the face as “neutral” and considered the gesture to be unimodal, allowing us to compare it to its use as part of a combination with either of the two facial expressions. Facial expressions without accompanying gestures were not coded, as chimpanzee faces were often not or not fully visible, so that we could not reliably compare the use of single facial expressions with their combinations with a gesture. If a gesture occurred and the facial expression was not fully visible, we coded this as a combination with “face not visible.” These combinations, however, were not included in the final analyses.

For each event, when individuals used either one of the two gesture types in isolation or in a combination with one co-occurring facial expression, we coded the individual producing the gesture as “initiator,” and the individual the signal was directed to as “recipient.” The social contexts in which a gesture or combination was used were categorized as positive/affiliative events (“pos”) or as negative/agonistic (“neg”) events. Positive events included contexts like greeting, grooming or mother-infant interactions and neg events included contexts like fighting over food or other resources, rank conflicts or harassment.

The behavior of the recipient was coded either as “affiliative” (approaching), including behaviors like embracing, the initiation of grooming, and the initiation of play, or “non-affiliative” (avoidant and/or ignoring), including behaviors like defensive reactions (e.g., hitting, pushing), chasing or turning away from the initiator. If a behavior could not be identified (e.g., because of poor visibility), it was coded as “not visible” and eliminated from analysis.

### Inter-coder reliability

To assess inter-coder reliability, a second independent person coded 10% of the videos (6.5 h). For each variable, Cohen’s kappa was calculated, resulting in excellent levels of coder agreement for all variables (Fleiss, 1981), ranging between 0.84 and 0.94 (mean = 0.88; identity of the individuals: 0.84, gesture: 0.94, facial expression: 0.87, response behavior: 0.88, context: 0.86).

### Statistics

To analyze the data, we used generalized linear mixed-effects models (GLMM) to avoid pseudoreplication by accounting for multiple responses measured for the different individuals and to handle non-normally distributed data by using link functions and exponential family distributions (e.g., binomial). We applied the GLMM with a binomial error structure to test our hypothesis regarding the differential usage of single gestures and their combinations with facial expressions by analyzing the context in which these were used and the combined effect of these factors on the response behavior of the recipient. We used the response of the recipient as response variable, which was binarily coded as affiliative or non-affiliative. As the factors gesture, facial expression and context have multiple levels, we included the combination of the three factors per data point as a random effect into the model (see Raw Data file provided with this manuscript for the model formula). To test for a possible effect of signal combination and context on the recipient’s response, we compared two models, one with the combination included and one without, by using a likelihood ratio test (LRT; Barr et al., 2013; Dobson & Barnett, 2008), available as R function “anova,” package “stats.”

Additionally, we included the sex of the initiator and recipient, the rank relationship of the interacting individuals (whether the single signal or combination was directed toward a lower or higher-ranking individual) and the chimpanzee group (1 vs. 2), as well as the year of data collection (2015 vs. 2016) to control for an influence of the changed group composition. Furthermore, to control for pseudo-replication due to repeated sampling from the same individual, we included the IDs of the dyad in addition to the initiator and recipient as random factors into the model (Crawley, 2002). We fitted the model in R Development Core Team (2017) using the function “glmer” from the R package “lme4” (Bates et al., 2015). As we were mainly interested in the random effect “signal combination,” we checked the assumption of a normally distributed variance of the random effects and found no violation. To secure model validity with regard to the fixed effects, we additionally checked the model stability by excluding each level of the random effects one at a time from the data set and compared the model estimates derived for these data with those derived for the full data set. This indicated no influential cases. We also calculated variance inflation factors (VIF; Field, 2005) using the function “vif” of the R-package “car” (Fox & Weisberg, 2018) applied to a standard linear model excluding the random effects, which revealed no collinearity in the data (largest VIF = 1.497). We considered *p*-values ≤ 0.05 to be significant and *p*-values > 0.05 and < 0.1 as trend.

## Results

The data set consisted of a total of 252 events. From these, 103 events (41%) were observed in group 1 and 149 events (59%) in group 2. From the total of 252 instances, 70 (28%) gestures occurred with a neutral face. The remaining 182 instances (72%) represented combinations of a gesture with a facial expression (31 bared teeth facial expressions; 77 funneled lip expressions). From all events, in 142 (62 in group 1 and 80 in group 2) instances (56%) the SG gesture was used and in 110 (41 in group 1 and 69 in group 2) instances (44%) the BG gesture was used.

**Figure 3:**
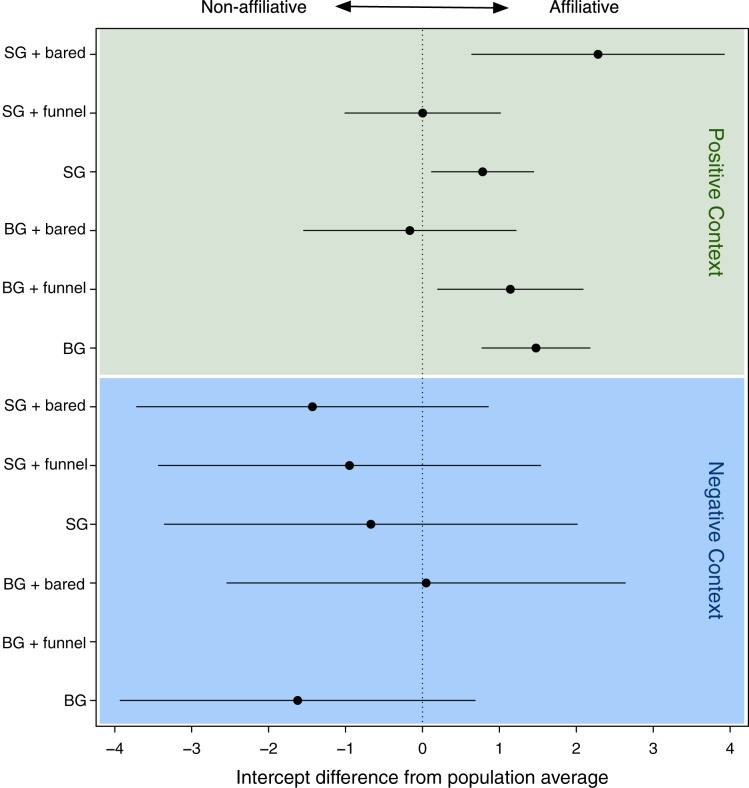
Confidence intervals of the deviations of the intercepts from the population mean for the levels of the random effect (combination of face, gesture and context). Especially for the negative context, we found large CI’s reflecting the rare data points within this context. SG, stretched arm gesture; BG, bent arm gesture; bared, bared teeth display; funnel, funneled lip face; neu, neutral face; pos, positive context; neg, negative context. BG_hoot_neg did not occur.

The GLMM analysis revealed a significant effect of the face-gesture-context combinations (LRT model with random effect vs. model without): χ² = 8.93, d*f* = 1, *p* = 0.003) on the recipient’s response (affiliation vs. non-affiliation). In order to interpret differences among the types of face-gesture-context combinations, we used the intercepts of the levels of the random effect (i.e., the face-gesture-context combinations), which indicated the effect on the recipient’s response, with a positive intercept indicating an affiliative response, and a negative intercept a non-affiliative response (Fig. 3; Table 1).

**Table 1:** Values of the intercepts and confidence intervals of the deviations of the intercept from the population mean for the levels of the random effect.

Combinations	Intercepts	CIs	Lower limit	Upper limit
AS_bared_pos	2.283	1.641	0.642	3.923
AS_bared_neg	−1.430	2.285	−3.715	0.855
OG_bared_pos	−0.164	1.380	−1.544	1.216
OG_bared_neg	0.048	2.589	−2.541	2.637
AS_funnel_pos	0.003	1.010	−1.007	1.013
AS_funnel_neg	−0.948	2.483	−3.432	1.535
OG_funnel_pos	1.143	0.945	0.198	2.087
OG_hoot_neg	NA	NA	NA	NA
AS_neu_pos	0.782	0.664	0.119	1.446
AS_neu_neg	−0.671	2.683	−3.353	2.012
OG_neu_pos	1.476	0.701	0.775	2.177
OG_neu_neg	−1.622	2.306	−3.928	0.684

**Note:**

BG_hoot_neg did not occur.

Figure 1 shows that when any signal combination was used in positive contexts (green area in Fig. 3), the response behavior was mostly affiliative (positive values), whereas when used in negative contexts (see blue area in Fig. 3), the response was mostly non-affiliative (negative values). This is not surprising.

However, a closer look at each context revealed striking differences depending on the different signal combinations. Specifically, in the positive contexts, there were significant differences regarding the influence of signal combinations on the response behavior (see Table 2). For all signal combinations that were used in negative contexts, these differences were not significant (see blue area in Fig. 3). Therefore, in the following, we focus on the significant differences in positive contexts only.

**Table 2:** Summary of the results.

Gesture	Face	Response
SG	–	Affiliation
SG	Bared teeth	Affiliation++
SG	Funnelled lip	No bias
BG	–	Affiliation
BG	Bared teeth	No bias
BG	Funnelled lip	Affiliation

**Note:**

Response behavior toward the different gesture + face combinations in positive contexts.

In general, when used as single gestures, both gestures (SG and BG) elicited affiliative responses (see Table 2). However, when combined with the two observed facial expressions, the response of the recipient was modulated in two different ways, depending on the type of signal combination: First, the unimodal SG_neu elicited an affiliative response in positive contexts, and when combined with the bared teeth facial expression (SG_bared), this tendency of an affiliative response increased. Thus, there was a stronger bias toward an affiliative response when the gesture was combined with the bared teeth facial expression compared to when it was used as unimodal gesture. Second, while the unimodal bent gesture with a neutral face (BG_neu) elicited an affiliative response when used in positive contexts, there was no bias toward either an affiliative or non-affiliative response when this gesture was combined with the bared teeth facial expression (BG_bared). Thus, while the unimodal gesture had an affiliative effect, this effect disappeared when this gesture was combined with the facial expression. The same pattern was observed for the unimodal SG gesture and when it was combined with the funneled lip face: unimodal usage led to an affiliative response, while there was no bias toward either an affiliative or non-affiliative response to the combination with the funneled lip face (SG_funnel). The most coherent result was the differential responses to each gesture, with and without the bared teeth face, and we provide our interpretation of this finding in the next section.

## Discussion

The aim of this study was to investigate whether there is evidence for compositional structures in chimpanzees’ visual communication, such that the combinations of a gesture with a facial expression and context of usage had a differential effect on the response behavior of the recipient. Our main finding is that facial expressions, when combined with a particular gesture, seem to have an augmentative effect on the recipients’ response. Thus, we show that the face and gesture are separable and recombinable, and that combinations get different responses than gestures alone.

However, this augmentative effect was evident in positive contexts only, and it differed depending on the gesture type. Thus, while both gestures elicited an affiliative response when used in isolation, their combinations with the bared teeth facial expression modulated the recipient’s response in different ways. For the SG, combinations with the bared teeth facial expression increased the probability of an affiliative response. For the BG, however, the bias toward an affiliative response disappeared when it was combined with the same bared teeth face. In other words, while both manual gestures produced a similar outcome when used alone, the combination with the bared teeth face elicited different responses in the recipient and either enhanced or decreased the bias toward affiliative behavior, depending on which gesture type was used. Importantly, only the bared teeth face showed these effects; gesture combinations with the funneled face did not alter the recipient’s behavior in similar ways. Furthermore, we only found augmentative effects in positive, but not negative contexts.

From our data, it is very difficult to make any conclusive statements regarding why we did not observe these effects in negative contexts. One possibility is that negative contexts might represent evolutionarily more urgent events in the sense that they might reduce an individual’s fitness (e.g., aggressive interactions, detecting and avoiding predators, etc.). Therefore, it is less efficient to use more flexible and complex multimodal communication (Tomasello & Zuberbühler, 2002), while unimodal signals, probably in the vocal domain (e.g., alarm calls), could be more appropriate and efficient in such negative interactions.

Our findings are not incompatible with those from existing studies on chimpanzees and different monkey species, although it is important to highlight that comparisons across studies are often difficult, since different methodological approaches are used, as we will explain. In what follows, we start with combinations in the vocal modality alone, first comparing our findings with a study claiming compositionality in a particular type of combination of vocal signals. We then go on to interpret other research on vocal signal combinations in the light of our findings, followed by research on multimodal combinations. Finally, we elucidate how our evidence for recombination of signals with different effects—which we refer to as “componentiality”—is a stepping stone for compositionality. We conclude by noting limitations of our study and offering suggestions for future research.

### A vocal “suffix”

A study using a unimodal approach on the vocalizations of wild Campbell’s monkeys showed that they may modify their “hok” call, which is a predator-specific alarm call uttered in response to the discovery of eagles. The modification renders a “hok-coo” call, which is a more general alarm call given in response to some kind of arboreal disturbance (Arnold & Zuberbühler, 2006; Ouattara, Lemasson & Zuberbühler, 2009b). A similar pattern was found for their leopard-specific “krak” call, which they transform into a more general “krak-oo” alarm call. Ouattara, Lemasson & Zuberbühler (2009a) suggested that adding the “-oo” element alters the meaning of highly specific alarm calls and went so far as to compare this to suffixation in human language. Similarly, several studies on different species of monkeys showed that they produce sequences containing combinations of different call types, which may convey different information than the individual call types that comprise them (Arnold & Zuberbühler, 2006; Ouattara, Lemasson & Zuberbühler, 2009b).

In light of these findings from vocal research, one could ask whether the combination of an extended arm gesture with a bared teeth facial expression represents a similar pattern as the combination of the “krak” call with the “-oo” element. That is, is it possible that at least some facial expressions, like the bared teeth-face, have a similar function to that of the “-oo” element, altering the meaning of the co-occurring signal?

In combination with the SG, the bared teeth signal can be interpreted as somehow augmentative, like “-oo.” However, these combinations have somewhat different properties than call +“-oo.” The first relates to context. While “-oo” was prompted by a more general threatening context than was the call without this “suffix,” in our data, the generally affiliative context was not different with or without adding the facial expression to the gesture. The second relates to the response. While no noticeable difference in response was described when the “-oo” call was added, in our data, adding the facial expression to the gesture was more likely to elicit a positive response, thus interpreted as performing a modifying function.

A third difference is that “-oo” calls did not occur in isolation, while the bared teeth face can be used without any co-occurring signals. If we continue with the language comparison, this is not a problem, as we can compare this third difference to free vs. bound morphemes, such as “not” (free) vs. “un-” or “-ness” (bound) in English, both of which enter into compositional structures. Thus, none of these differences rules out a compositional interpretation of the SG + bared teeth face combination. Therefore, while we do not have directly comparable phenomena, in both the monkey calls and the chimpanzee signals, the addition of a call or a facial expression makes a change in the signal which is not random. In both cases, combining two signals results in a difference, either in the motivation for, or the effect of the combined signal.

### Other studies of vocal signal combinations

Townsend et al. (2018) suggest that compositional structures might be more likely to be found in the vocal domain. However, we remain agnostic on this issue. A direct comparison with the current findings is difficult, as these vocal studies (Coye et al., 2015; Engesser, Ridley & Townsend, 2016; Ouattara, Lemasson & Zuberbühler, 2009a) differ from our study in several ways. First, vocal studies focus on functionally referential vocalizations, which are used in response to very specific events in the monkeys’ environment, and therefore have very specific meanings. Gestures, however, are much less specific, although it has been claimed that at least some of them have specific meanings (Hobaiter & Byrne, 2014; but see Liebal & Oña, 2018). Second, the vocal studies by Arnold & Zuberbühler (2006) and Ouattara, Lemasson & Zuberbühler (2009a, 2009b) focus on one modality only, raising the possibility that the monkeys’ communication may be even more complex, if other signal types or contextual information were taken into consideration (Wheeler & Fischer, 2012). Third, these studies investigated sequential structures, consisting of different call types combined one after the other, while our study focused on simultaneous combinations. Taken together, this underscores the difficulty of comparing studies and interpreting the significance of complex, multimodal signals in primate communication.

### Multimodal combinations

Only few studies have used a multimodal approach and considered more than one signal type. For example, Wilke et al. (2017) examined wild chimpanzees’ response rates to single gestures and to vocalizations (grunts) in comparison to gesture-grunt combinations. As in our study, they found that multimodal combinations were only rarely produced in comparison to single gestures or vocalizations. Chimpanzees responded more often to gesture-grunt combinations compared to when the grunt was produced alone, but not compared to the single gesture. This means that the gesture alone seems sufficient to elicit a response, which resembles our findings for combinations of the offer gesture with the funneled face, which was as likely to elicit an affiliative response as the gesture alone. However, if the bent wrist gesture was combined with the bared teeth face, this bias toward affiliative responses vanished. Importantly, unlike Wilke et al. (2017), who focused on response rates, we investigated how combinations influence response types. Furthermore, Wilke et al. (2017) did not consider facial expressions, but considered chimpanzees’ gestural repertoires including body postures, while we focused on specific gesture types. Together, these comparisons with other unimodal and multimodal studies show that it is a challenge to estimate the existence and effect of compositional structures in primate communication, as such studies are still scarce and they vary widely with regard to their methodological approaches.

### Componentiality as a stepping stone to compositionality

The fact that the bared teeth face has a different effect on the BG than on the SG is intriguing. In order to have a compositional system, the first prerequisite, before attributing particular meanings to signals, is to identify separate components that can occur in different combinations—in other words, to identify componentiality in the system. By no means can componentiality be taken for granted. For example, certain chimpanzee facial expressions by definition occur with particular vocalizations (e.g., the hoot face with the hoot vocalization). If this is true, then, although we can identify the components (vocal and facial), such combinations are not componential. Our results do demonstrate componentiality. The fact that the bared teeth face can combine either with the SG or with BG shows that the gestures/faces are identifiable components. That different combinations trigger different responses confirms that the system is componential. It is not possible to conceive of compositionality without the necessary step of isolating and recombining components, regardless of meaning. Thus, our study reveals componentiality, a necessary prerequisite for compositionality, a prerequisite which has not been identified as such in previous work. This discovery in itself offers an advance in our understanding of the evolution of compositional systems.

### Limitations and future research

One limitation of the current study is our focus on *specific* behavioral contexts (interactions with high-ranking males around feeding times) determined by the observational conditions in the sanctuary. We also included only two gestures in our analyses, although we identified additional gesture types, which could not be considered because of their low frequencies of occurrence in these contexts. Furthermore, the coding of facial expressions was often not possible due to limited visibility of the face (e.g., individuals turning the head while gesturing). Therefore, we were not able to include matched control situations for unimodal facial expression events (Wilke et al., 2017), where a gesture-face combination is compared to a matched corresponding unimodal facial expression with regard to the interacting individuals, the context and the recipients’ response. The amount and structure of our data set did not facilitate a fine-tuned analysis regarding the behavioral responses of the recipient. Thus, the biggest challenge for identifying compositional structures in chimpanzees and other primates is to produce a sufficiently large data set, consisting of high-quality video footage, which enables researchers to code different signal types in different behavioral contexts.

Investigating vocalizations in addition to the gesture-face combinations was thwarted by interference of many vocalizations at a time from other bystanding individuals when a dyadic event was recorded. Thus, we could not reliably identify the source of a vocalization, nor could we obtain recordings of sufficient quality to conduct an acoustic analysis. The number of events for which we could assign a vocalization to a given individual with sufficient certainty, and reliably analyze the acoustic signal, was too low to include in our analyses.

Future studies should tackle this issue and, ideally, should combine data from several researchers from different research sites. Such a comprehensive data set, together with a multimodal approach jointly considering gestures, facial expressions and vocalizations, will enable us to capture the complexity of primate communication. In this way, we can learn more about the building blocks of compositional communicative systems, and determine whether or not compositionality in this domain can be unequivocally attributed to primates other than humans.

## Conclusion

Compositionality is the remarkable ability to combine and recombine a finite number of meaningful units to produce the open-ended, flexible communication system characteristic of human language. It is perhaps not surprising that our study of visual chimpanzee facial expressions and manual gestures did not find a compositional system of this kind, in which each facial expression and each manual gesture contributes a constant meaning or effect when recombined with other expressions or gestures. But neither were the facial and manual components that we tracked conveyed and interpreted holistically, i.e., as indivisible wholes. Intriguingly, we found something in between—the ability to recombine parts of a visual display resulting in different effects on conspecifics in particular contexts—a property that we refer to as componentiality. In our study, the bared teeth component on the face had differential effects on the two manual gesture components investigated. We reason that compositionality could not have evolved without the capacity first to isolate components and recombine them, with different communicative effects. Therefore, we see componentiality as a necessary stepping stone to compositionality. We hope that the methods and findings reported here can be extended to a larger repertoire of visual signals as well as vocalizations in chimpanzees and related species, in order to confirm and further explore this important communicative device.

## Supplemental Information

10.7717/peerj.7623/supp-1 Supplemental Information 1 Raw data.

## Additional Information and Declarations

### Data Availability

The following information was supplied regarding data availability: Data is available at Figshare: Oña, Linda (2019): Compositionality_data. figshare. Dataset. https://doi.org/10.6084/m9.figshare.9192509.v1.
